# Guided Superficial Enhanced Fluid Fat Injection (SEFFI) Procedures for Facial Rejuvenation: An Italian Multicenter Retrospective Case Report

**DOI:** 10.3390/clinpract13040085

**Published:** 2023-08-08

**Authors:** Alessandro Gennai, Monica Baldessin, Fabrizio Melfa, Bruno Bovani, Alessandra Camporese, Barbara Claysset, Mattia Colli, Alberto Diaspro, Rosalba Russo, Placido Strano, Daniele Bollero, Guido Capparè, Alessandro Casadei, Giovanni Gallo, Domenico Piccolo, Giovanni Salti, Piero Tesauro

**Affiliations:** 1 Studio Gennai, 40122 Bologna, Italy; 2 Studio MB, 31100 Treviso, Italy; monica.baldessin@gmail.com; 3 Mediaging Clinic Center, 90143 Palermo, Italy; dottormelfa@gmail.com; 4 Centro di Chirurgia Ambulatoriale Esculapio, 06124 Perugia, Italy; bovanibruno@gmail.com; 5 Studio Camporese, 35010 Cadoneghe, Italy; alessandracamporese@gmail.com; 6 BC Medical, 40137 Bologna, Italy; babyclaysset@gmail.com; 7 Centro Podgora 7, 20122 Milano, Italy; mattiacolli@shbclinic.com; 8 Rigeneralab Centre for Regenerative Medicine Torino, 10134 Torino, Italy; info@albertodiaspro.com; 9 Estemed, 41122 Modena, Italy; pol.estemed@gmail.com; 10 Beautè Medical, 33170 Pordenone, Italy; studio.strano@gmail.com; 11 CTO Hospital, 10126 Torino, Italy; info@danielebollero.it; 12 Studio Medico Strozzieri, 64021 Giulianova, Italy; ricercaguido@alice.it; 13 Ambulatorio Casadei, 30174 Venezia-Mestre, Italy; ale.casadei@hotmail.it; 14 Centro Mediestelaser, 92024 Canicattì, Italy; g.gallo@mediestelaser.it; 15 Skin Center, 65121 Pescara, Italy; domenico.piccolo.skincenters@gmail.com; 16 Medlight Institute, 50144 Firenze, Italy; giovannisalti@gmail.com; 17 Poliambulatorio Gioia, 20124 Milano, Italy; pierotesauro@gmail.com

**Keywords:** facial rejuvenation, stromal vascular fraction, adipose-derived mesenchymal stem cells, autologous adipose tissue graft, superficial enhanced fluid fat injection, clinical regeneration applications

## Abstract

Background: The aging process starts in the center of the face, in the periocular region and around the mouth, with a combination of volume loss, tissue descent, deepened wrinkles, and the loss of skin structure and quality. Recently, several studies have demonstrated the efficacy of therapies based on autologous adipose tissue grafting, which leverages the properties of stromal vascular fraction (SVF) and adipose-derived mesenchymal stem cells (ADSCs) to accelerate the regenerative processes of the skin. This study aims to verify the ability of guided superficial enhanced fluid fat injection (SEFFI) in the facial area to correct volume loss and skin aging, proving that this standardized procedure has a very low rate of complications. Methods: We retrospectively collected data from 2365 procedures performed in Italian centers between 2019 and 2021. Guided SEFFI was performed alone or combined with cosmetic treatments, including the use of hyaluronic acid filler, suspension threads, synthetic calcium hydroxylapatite, botulin toxin, and microneedling. Results: guided SEFFI was used alone in more than 60% of the patients and in all facial areas. In about one-tenth of the patients, guided SEFFI was combined with a botulin toxin treatment or hyaluronic acid filling. Other procedures were used more rarely. Ecchymosis in the donor or injection sites was the most frequent adverse event but was only observed in 14.2% and 38.6% of the patients, respectively. Conclusions: The guided SEFFI technique is standardized and minimally invasive, leading to very few complications. It constitutes a promising antiaging medical treatment that combines effectiveness, safety, and simplicity.

## 1. Introduction

Aging is a natural and biological occurrence. In middle-aged patients from 45 to 55 years old, skin atrophy and volume loss, which are mostly associated with variable degrees of tissue ptosis, are the most significant factors at work in aging [1].

As the skin ages, the dysregulation of the stem cell population, which usually helps to repair damaged tissue, may occur [2]. In addition to intrinsic (chronologic) aging, the skin may also experience an extrinsic aging process due to exposure to various environmental stressors, such as ultraviolet (UV) radiation, trauma, chemicals, smoking, and reactive oxidative species [3,4]. All of these factors lead to an accumulation of DNA damage with the consequent impairment of protein maturation, cell function, and normal physiology [2].

In the periocular region, the thinning of the eyebrows and the deepening of the superior eyelid sulcus, along with infraorbital hollows, are worsened by midfacial descent [5,6,7,8], whereas in the perioral region, the worsening of the nasolabial folds, marionette lines, and lip atrophy occur. Clinically, aging manifests as fine lines and wrinkles, a loss of elasticity, dyschromia, epidermal thinning, and increased coarseness [3,9]. The volumetric loss of contours and angularity in the face are also visible due to tissue atrophy within the deep layers of the skin [10,11]. Regenerative therapy exploits the properties of the cells of the stromal vascular fraction (SVF) of the adipose tissue to counteract these aging-associated changes.

However, regenerative medicine currently resorts to injecting adipose-derived stem cells (ADSCs) because of their characteristics and availability [12,13,14,15,16].

In recent years, several studies have shown the efficacy of therapies based on the autologous grafting of adult mesenchymal stem cells to accelerate the healing and regenerative processes of the skin and mesenchymal tissues. Adult mesenchymal stem cells are pluripotent adult progenitor cells derived from embryonic connective tissue. ADSCs exhibit regenerative activity due to their intrinsic ability to transform into cells of the mesenchymal and endothelial lines, promoting tissue repair [17,18].

The stromal vascular fraction (SVF) of adipose tissue contains many cells, creating interrelated cell populations including adipocyte progenitors, pericytes, endothelial progenitor cells, and transit-amplifying cells [19]. Adipose-derived stem cells (ADSCs) can secrete bioactive molecules that stimulate angiogenesis and have antifibrotic, antiapoptotic, and immunomodulatory properties [20,21,22,23].

Moreover, SVF/ASCs induce the secretion of cytokines and growth factors, which promote angiogenesis and, thus, the revascularization of fat grafts [24,25].

Such characteristics of SVF/ASCs may account for some effects observed after adipose tissue implantation, such as improved skin trophism, the accelerated closure of complex wounds or ulcers, and the enhancement of the skin’s appearance after damage from radiotherapy [26,27].

In 2015, one of the authors [28] standardized a new tissue graft technique called superficial enhanced fluid fat injection (SEFFI) to achieve skin enhancement and the restoration of facial volume.

This technique is used to harvest adipose tissue using a microcannula with small side portholes (0.3–0.5 and 0.8) in a superficial adipose tissue layer (SAT) [28,29] with the aim of grafting adipose tissue, including SVF cells and the ADSCs contained therein.

Since 2015 [17,28,29,30,31,32,33], several studies have been published that prove that by using a special cannula with tiny side portholes, the adipose tissue can be harvested so as to select small cellular clusters that do not require any manipulation to reduce the clusters’ dimensions and fluidify the tissue.

The absence of substantial manipulation guarantees the maximum viability and stemness of the harvested tissue [34,35].

Although SEFFI is a minimally invasive technique, it still requires surgical skill to collect the adipose tissue through a small skin hole and in the superficial subcutaneous plane.

In 2020, A. Gennai et al. [36] published a simplified guided SEFFI procedure that allows for the harvesting of tissue in a safe and effective plane, even without major surgical skills.

In this study, we present data retrospectively collected by ten physicians who carried out more than 2300 guided SEFFI procedures in various anatomic locations of the face and for diverse indications between 2019 and 2021.

## 2. Materials and Methods

### 2.1. Adipose Tissue Harvesting Procedure

This study is a retrospective analysis of data obtained from 2365 healthy patients, of whom 2123 were females aged, on average, 49.2 years old (range: 38–65 years), and 242 were males aged, on average, 47.0 years old (range: 41–53 years). Patients were treated in 10 different professional centers for aesthetic physicians and surgeons, with the guided SEFFI technique performed using a SEFFILLER™ medical device between 2019 and 2021. No gender or age exclusion criteria were applied in this retrospective study.

The follow-up of the cases was between 4 and 6 months depending on the center.

Adipose tissue was harvested from patients after informed consent was obtained under the Declaration of Helsinki guidelines. All procedures were performed in private Italian medical centers, so Institutional Review Board approval was not required.

The guided SEFFI harvesting technique was previously described in [36].

**Figure 1:**
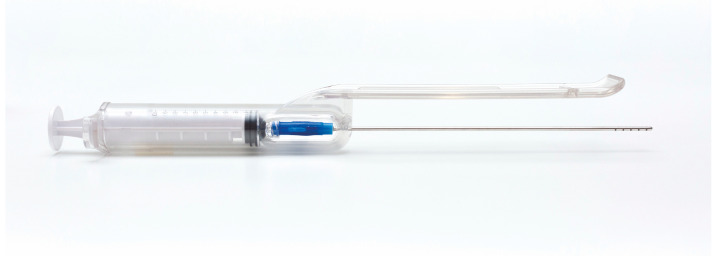
The guide with syringe and cannula. The guide is meant to standardize the adipose tissue harvesting procedure.

Briefly, the procedure was performed under local anesthesia using a microperforated cannula with 0.8 mm side port holes mounted inside the unique patented guide (Figure 1).

**Figure 2:**
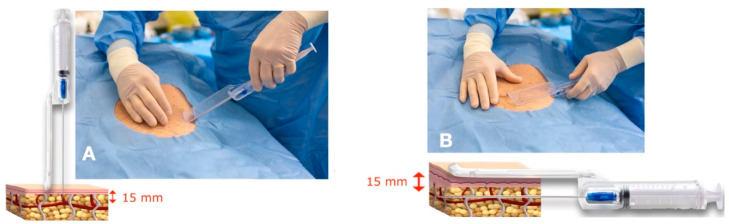
The guide allows for the cannula to penetrate no more than 15 mm under the skin and to collect superficial adipose tissue (SAT) at a 15 mm depth. (**A**) Penetration of the tip of the cannula at a 15 mm depth. The blade of the guide avoids deeper penetration of the cannula. (**B**) The blade of the guide touches the skin, and the cannula collects the superficial adipose tissue (SAT) at a 15 mm depth.

Superficial adipose tissue (SAT) was collected using a gentle back-and-forth fan movement throughout the sampling area. The main harvesting areas were the abdomen, trochanter, and hip, among other areas. The guide was used to standardize the procedure and guarantee that tunneling was performed in the subcutaneous adipose tissue adjacent to the dermis (SAT), which is particularly rich in mesenchymal and vascular stem cells, and in the same plane for all samples [20,37] (Figure 2).

**Figure 3:**
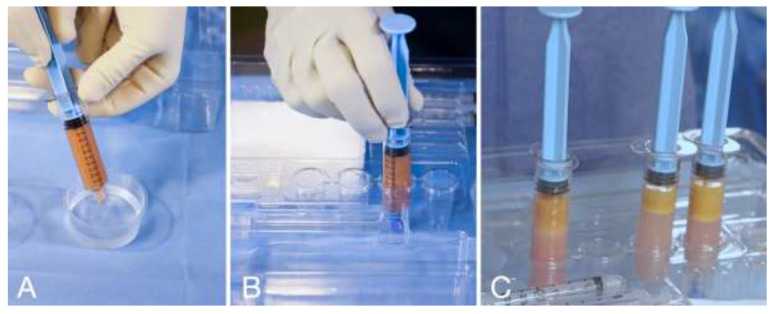
(**A**) Reservoir syringe filled with saline solution and capped. (**B**) Reservoir syringe placed in the syringe holder in vertical position for decanting. (**C**) After 5 (five) minutes of decanting, the tissue is separated from the washing liquid; the washing liquid will be discharged.

The harvested adipose tissue was transferred into the reservoir syringes. The reservoir syringes were filled up with a saline solution, cupped, and left in a vertical position for 5 (five) minutes to allow for the content to stratify via the force of gravity in the following three layers: oil, tissue, and washing liquid in the bottom of the reservoir syringe (Figure 3).

The washing liquid was discharged, and the tissue was ready to be prepared for the injection. The rationale for washing tissue with saline solution is to remove blood and lidocaine from the tissue. Moreover, a study showed that decantation is associated with maximum viability of adipocytes; thus, this method is the standard of maximum viability [37].

**Figure 4:**
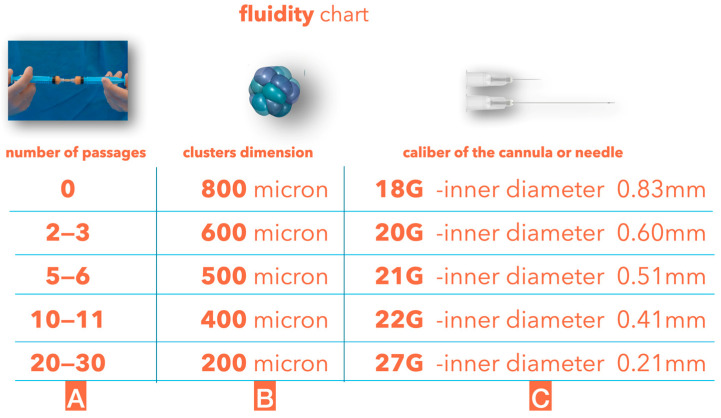
(**A**) Number of tissue passages from one syringe to another through the transfer mechanism without any filter. (**B**) Average cluster dimensions after the passages. (**C**) Average dimensions of the cannula or needle used to inject the tissue.

Different areas of the face have different skin and subcutaneous thicknesses, so it is essential to inject the proper fluidity of tissue superficially without any risk of visibility and lumpiness. The clusters’ dimensions can be further reduced to obtain different degrees of fluidity; with the guided SEFFI technique, such a reduction can be achieved using an emulsification procedure by connecting two 10 mL syringes through a transfer mechanism provided in the device and performing passages of tissue from one syringe to the other (Figure 4).

The fresh microfragmented adipose tissue obtained using the guided SEFFI technique does not require any further substantial manipulation due to the small dimensions of the clusters harvested using the SEFFI cannula with 0.8 mm side port holes; light manipulation (passaging from one syringe to another) should only be performed if the fluidity needs to be increased to treat very delicate areas of the face.

The learning curve of the guided SEFFI technique is very shallow, and the device is designed to perform the procedure in a simple, safe, effective, and standardized way for both surgeons and physicians. The device is guided, disposable, and all in one; although the procedure is simple, all surgeons and doctors who have performed this procedure have attended a training course offered by the company (Seffiline Academy).

### 2.2. Guided SEFFI Single Treatment

**Figure 5:**
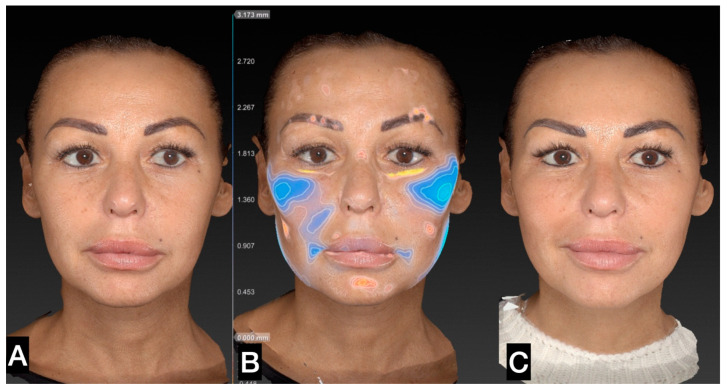
(**A**) A 46-year-old woman prior to guided SEFFI single treatment; (**B**) following treatment (blue indicates treated areas); and (**C**) 45 days post treatment.

**Figure 6:**
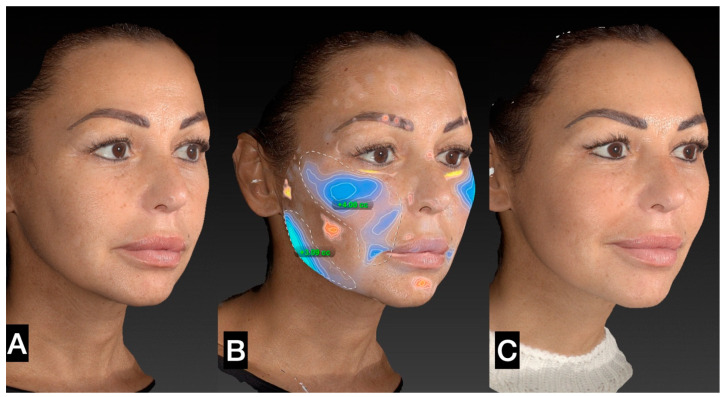
(**A**) Right side prior to SEFFI single treatment; (**B**) treated area and amount of tissue injected; (**C**) 45 days post treatment.

**Figure 7:**
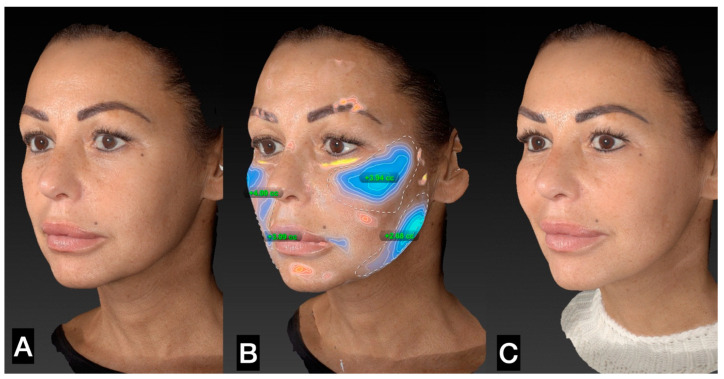
(**A**) Left side prior to guided SEFFI single treatment; (**B**) treated area and amount of tissue injected; (**C**) 45 days post treatment.

**Figure 8:**
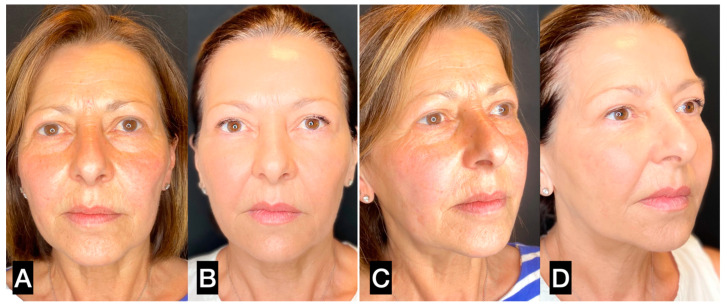
(**A**,**C**) A 56-year-old woman prior to guided SEFFI single treatment; (**B**,**D**) 4 months post treatment.

**Figure 9:**
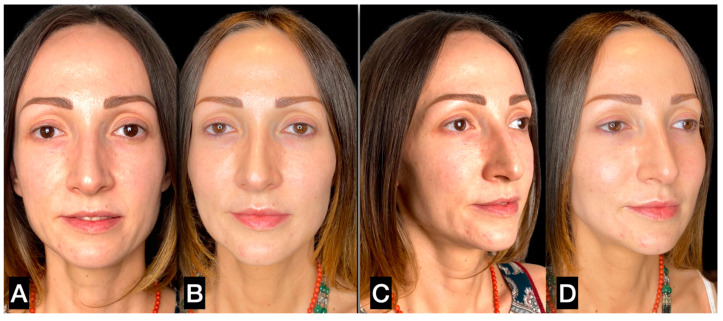
(**A**,**C**) A 31-year-old woman prior to guided SEFFI single treatment; (**B**,**D**) 6 months post treatment.

The tissue was transferred to smaller syringes. The injection is performed using a 20 G or 21 G microcannula in the very superficial subcutaneous layer. The goal of the treatment is “to sow” microfragmented adipose tissue in the very superficial subcutaneous layer (Figure 5, Figure 6, Figure 7, Figure 8 and Figure 9).

**Figure 10:**
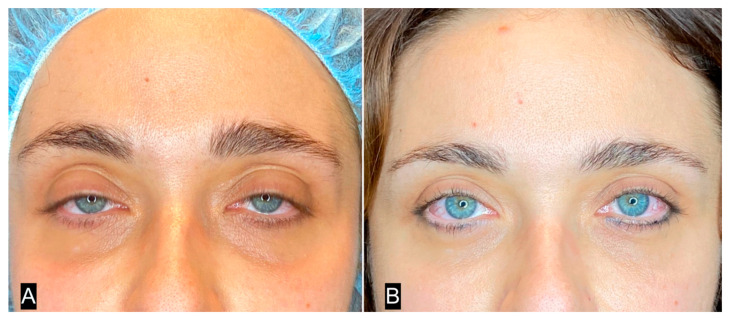
(**A**) A 32-year-old woman with deep inferior sulcus in the eyelid; (**B**) 3 months after single SEFFI guided treatment.

**Figure 11:**
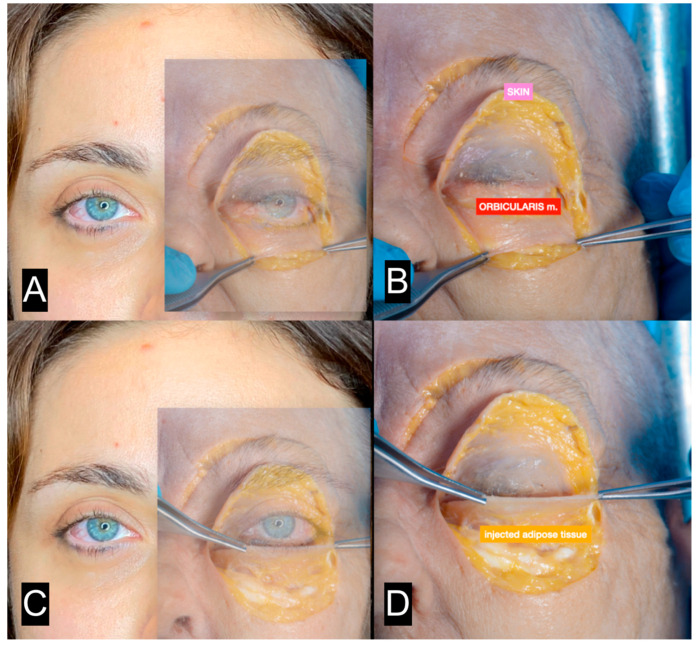
(**A**) Patient shown in Figure 10A with superimposed transparent image from cadaver dissection (the skin is elevated). (**B**) Cadaver dissection. The orbicularis muscle is exposed. (**C**) Patient shown in Figure 10A with superimposed transparency image from cadaver dissection (the orbicularis muscle is elevated). (**D**) Cadaver dissection. The injected tissue is exposed.

The periocular area is the only area where the authors suggest injecting tissue in the submuscular plane [30,37,38] (Figure 10 and Figure 11). In this area, the skin is very thin, and the subcutaneous tissue is minimally represented. Furthermore, the skin adheres to the orbicularis muscle; therefore, the tissue must be injected under the muscle in order to avoid lumpiness or visibility.

### 2.3. Guided SEFFI and Hyaluronic Acid

**Figure 12:**
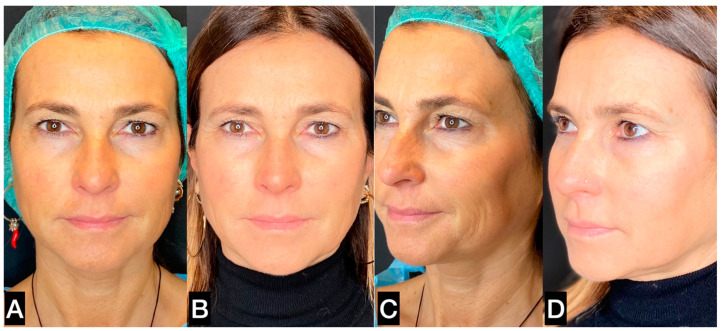
(**A**,**C**) A 43-year-old prior to treatment; (**B**,**D**) 6 months post treatment, in which 1 mL crosslinked hyaluronic acid (HA) was injected in the deep subcutaneous plane in each malar and zygomatic area, combined with 5 mL guided SEFFI injected in the superficial subcutaneous plane in the same area in the same session.

Combined guided SEFFI and hyaluronic acid (HA) treatments are performed to enhance the volume of the face in a particular area, such as the malar, zygomatic, and jawline areas [30]. In such cases, the HA injection is performed before the more profound guided SEFFI. A previous study proved that the combination of guided SEFFI and HA is available for the autologous ready-to-use treatment of human skin aging, which can be achieved without pretreatment of enzymes and yields a highly homogeneous MSC population. The combination of fresh tissue obtained with guided SEFFI procedure with HA product can be exploited to counteract the loss of volume and skin aging in the human face and body. Examples of critical aesthetic areas of aging are wrinkles around the mouth, loss of volume of the malar fat pad, tears through the periocular area, and hand and neck wrinkles. The non-expanded autologous fat product SEFFI with HA may pave the way for the development of novel approaches and paradigms for facial rejuvenation within the context of personalized autologous regenerative medicine [39,40,41] (Figure 12).

### 2.4. Guided SEFFI and Suspension Threads

**Figure 13:**
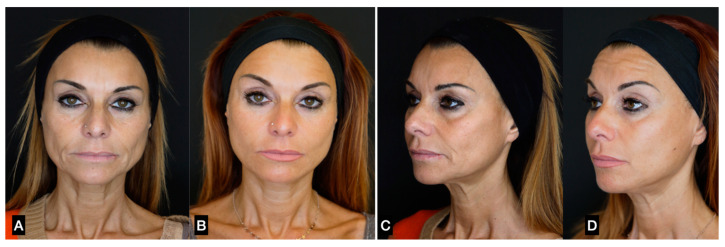
(**A**,**C**) A 47-year-old woman prior to treatment; (**B**,**D**) 6 months post treatment with suspension threads in the jawline combined with guided SEFFI injected in the malar–zygomatic and temporal areas.

The three main factors in facial aging are volume loss, skin aging, and tissue descent. The combination of guided SEFFI and suspension threads is a facial rejuvenation treatment meant to restore volume, regenerate the skin, and reposition the superficial tissue. When guided SEFFI and threads are applied in the same area, the guided SEFFI procedure must be completed first; after three weeks, the threads can be positioned. If the two treatments are applied in different areas (for example, guided SEFFI in the malar–zygomatic, perioral, and periocular areas and suspension threads in the jawline area), they can be performed during the same session. A retrospective analysis of 232 cases of patients seeking rejuvenation of the lower frame of the face (jowls and jawline) proved that the combination of these techniques is promising for medical treatment of mild to moderate facial laxity, with a fast recovery and a very low complication rate [42] (Figure 13).

### 2.5. Guided SEFFI and Microneedling

Photoaging and smoking are the leading causes of superficial wrinkles, particularly in the perioral area (“barcode wrinkles”). Skin microneedling is a technique that accelerates the process of skin regeneration through the creation of numerous microinjuries, which emerge when skin is deeply punctured with tiny needles. The procedure evokes various reactions, which can be divided into the following three major phases: inflammation, proliferation, and remodeling. Microneedling activates platelet growth factors, which are responsible for the stimulation of fibroblasts to produce collagen and elastin. Treatment can be performed using different devices, all equipped with needles of various lengths, as skin microneedling stimulates the synthesis of significant rebuilding and structural skin elements [43].

Several studies have analyzed in vitro actions of adipose-derived mesenchymal stem cells (AD-MSCs) against the effects of photoaging, thanks to their migratory activity, paracrine actions, and related in vivo–ex vivo outcomes. AD-MSCs act against skin photoaging in vitro via the activation of dermal fibroblast proliferation, antioxidant effects, and matrix metalloproteinase (MMP) reduction [44,45].

**Figure 14:**
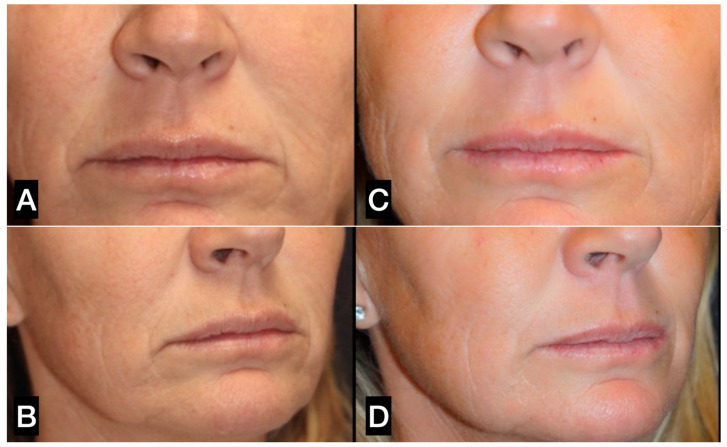
(**A**,**C**) A 49-year-old woman prior to treatment; (**B**,**D**) 12 months post perioral treatment with 2 guided SEFFI treatments (2 treatments 6 months apart, followed by 6 microneedling treatments).

In light of this evidence, the authors of this study proposed the combination of guided SEFFI and microneedling to treat superficial skin wrinkles, particularly in the perioral area and chin. The two treatments were combined in the same session; guided SEFFI was carried out as the first procedure, followed by microneedling treatments (5–8 treatments weekly) (Figure 14).

### 2.6. Guided SEFFI and Botulin Toxin

Gaze rejuvenation is crucial in face antiaging treatment, as the periocular area plays a fundamental role in the beauty and youthful aspect of the face. The main factors involved in gaze aging are dynamic wrinkles, the lowering of the eyebrow tails, the loss of volume, and skin aging of the periocular area.

Many authors have proven the effect of botulin toxin in both reducing periocular dynamic wrinkles and lifting the eyebrow tail [46,47,48].

Bernardini et al. [29] proved the regenerative and volumizing effects of the SEFFI technique in the periocular area.

Microfragmented adipose tissue injection involves fluid tissue. Injection was performed under the orbicularis muscle using a microcannula with a relatively slow retrograde technique. Before the tissue injection, an anesthetic solution with adrenaline (1: 100,000) was administrated under the orbicularis muscle. In this study, botulin toxin was administrated ten days before the guided SEFFI procedure.

### 2.7. Guided SEFFI and Carboxytherapy

Carboxytherapy is a medical technique during which a sterile gas, i.e., carbon dioxide, is injected into the subdermal tissue. The utilization of carbon dioxide injections has enhanced the practical relevance of carboxytherapy as a method for the management of multiple disorders. Carboxytherapy provides an attractive aesthetic option for skin rejuvenation, atrophic scars, striae distensae (stretch marks), cellulite–fibrolipodystrophy adhesion after liposuction, and certain types of alopecia. The proposed mechanisms of action include increasing the blood supply; reopening closed capillaries; dilating precapillary vessels; increasing oxygen levels through the Bohr effect, which consists of a shift of the oxygen dissociation curve to the right as a result of hypercapnia; decreasing oxygen consumption; increasing CO_2_ in tissues; and decreasing the affinity of hemoglobin for oxygen [49,50].

In light of these effects, the authors of this study combined tissue injection with carboxytherapy to combine the regenerative effect with the enhancement of tissue oxygenation. The two treatments were combined in the same section, and carboxytherapy was carried out (as a single procedure) 4 times every seven/ten days after the first combined treatment.

### 2.8. Guided SEFFI and Calcium Hydroxylapatite

Facial aging is a multifactorial and three-dimensional process involving volume loss, sagging, and skin alterations like elastosis. Every anatomical component of the face is affected. The combination of guided SEFFI and calcium hydroxyapatite has proven to be effective for a natural result of reharmonization of the face, especially in the scope of the duration of the results. The procedure involves (T0) the treatment of the temporal, malar, zygomatic, preauricular, mandibular angle, and marionette areas with 3 mL of calcium hydroxyapatite diluted in a ratio of 1:1 or with hyperdilution to 1:2 with a physiological solution to determine the neocollagenogenesis, as well as elastic production and angiogenesis, both of which are extensively covered in the literature [51,52,53].

**Figure 15:**
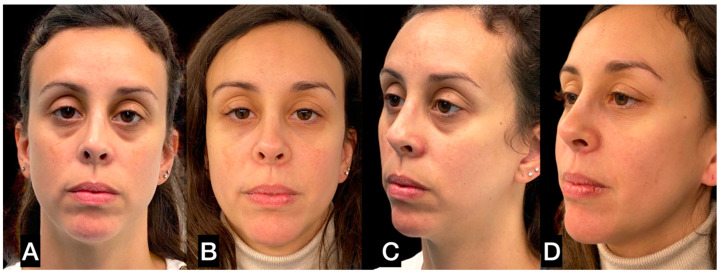
(**A**,**C**) A 39-year-old woman prior to treatment; (**B**,**D**) 6 months post treatment with 1 calcium hydroxylapatite filler (1.5 mL diluted in a 1:1 ratio with saline solution) treatment in the malar area followed by 18 mL guided SEFFI in the full face.

Implantation is accomplished using 38 mm 27 G or 50 mm 25 G cannulas on the supraperiosteal and subcutaneous or subdermal plane [54]. After two weeks (T1), the guided SEFFI treatment is performed using approximately 20–24 mL of adipose tissue. Implantation is performed on the subcutaneous and subdermal plane using 25 G or 22 G 50 mm cannulas. Integrating these two medical techniques leads to a natural and balanced result that lasts for a notable duration (Figure 15).

### 2.9. Guided SEFFI and Endolift

Guided SEFFI in combination with 1470 nm interstitial laser lifting with optical fiber is a perfect match for the treatment of facial aging.

Autologous regenerative therapy (ART) exploits the regenerative potential of microfragmented adipose tissue, which is naturally rich in stromal vascular fraction (SVF), progenitor cells (ADSCs), and growth factors (GFs).

The harvest of adipose tissue is carried out using a device that allows for ART to be performed on an outpatient basis in a simple, standardized, and safe way. The procedure includes the following three phases: guided extraction of the adipose tissue, decantation, and grafting—with maximum availability and cell vitality. The use of a cannula with microholes allows for the harvest of already fluid adipose tissue, which does not require further manipulation and can be injected into the SMAS and the dermal layers of the face using a microcannula (20–22 G).

Interstitial laser lifting at a wavelength of 1470 is a minimally invasive procedure that involves the use of 200–300 micron optical fibers (OF). Like a luminous path, the OF crosses the dermal layers, transmitting laser radiant energy in the near-infrared band. Through a process of redensification and dermal retraction, the procedure treats skin laxity and harmonizes and redefines the facial profile.

The two methods are combined during the same treatment session. Fan-shaped dermal retraction lines are created from the pretragal region towards the cheek and along the profile of the jaw (200 joules per side) with 200 or 300 micron optical fiber. Subsequently, a fluid fat graft is taken from the trochanteric region, which, after washing with a few cc of saline solution, is grafted in multiple layers on the full face using a 20 G cannula (8 cc per side).

**Figure 16:**
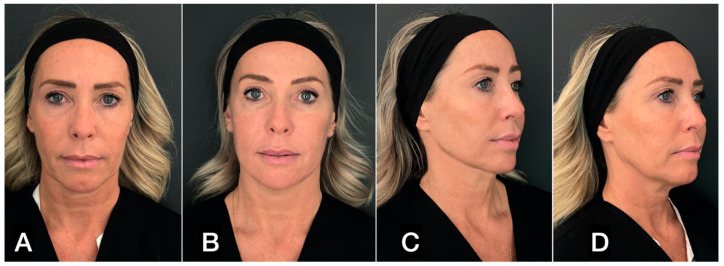
(**A**,**C**) A 50-year-old woman prior to treatment; (**B**,**D**) 4 months post treatment with 16 mL guided SEFFI in the full face followed by 300-micron flat-tip Endolift (Ton50; Toff75; pulse mode; 500 joule for the lower third of the face; and 150 joule for the neck).

The integration of the two methods promotes the metabolic functions of the extracellular matrix, improves vascularization, and amplifies the response of fibroblasts to neocollagenesis. The results, which become progressively visible after two weeks, show a global rejuvenation of the face in terms of skin tightening and skin quality (Figure 16).

## 3. Results

In this study, we analyzed the results obtained from a total of 2365 patients.

Table 1 reports the details of the fat harvesting sites and the volumes collected using guided SEFFI techniques. Fat tissue was gathered from the abdomen in more than 50% of the treated patients. The trochanteric area was also frequently selected (in about one-third of the patients). The procedure used to harvest fat from these two areas allowed us to obtain the same amount of tissue (27.1 mL). The hip was less frequently selected for the harvesting procedure, with a slightly lower yield (22.6 mL). The knee was also chosen as a collection site, although more rarely, as the amount of obtainable liquid is lower than that obtained from the other harvesting areas.

Table 1 also reports the details of the injection sites and volumes inoculated using the guided SEFFI techniques. Most patients (81.4%) were treated in more than one site of the face; in particular, the procedure involved the whole face in almost one-quarter of the patients (23.6%). The injection was performed in a single region of the face in only 18.6% of the patients, and the malar–zygomatic area was the most frequently selected site for a single administration (9.9% of patients).

Table 2 illustrates the types of aesthetic treatments performed using guided SEFFI alone or combined with other procedures. The guided SEFFI procedure was applied as a single procedure in more than 60% of the cases and involved all face areas. About one-tenth of the patients received a fat graft combined with a botulin toxin treatment or hyaluronic acid filling; fewer patients underwent other combined procedures.

Table 3 illustrates the adverse events that occurred at the donor and injection sites in the treated patients.

The rate of complications was low. Ecchymosis was the most common complication, occurring in both the donor and injection sites (14.2% and 38.6%, respectively).

In the donor site, other complications occurred infrequently, such as hematoma or skin irregularities lasting more than 20 days, which were experienced by only 1.3 and 0.2% of patients, respectively.

## 4. Discussion

In 2001, Coleman [55] studied a procedure that uses autologous fat tissue to restore facial volume. Autologous adipose tissue was believed to be the ideal filler, delivering natural filling in a safe and easy procedure [55].

However, this technique had limitations, such as unpredictable fat survival and a substantial risk of visible lumps. Consequently, many authors have focused on microfragmented adipose tissue grafts, which are naturally rich in SVF and ADSCs, as a valuable approach to the aesthetic rejuvenation treatment to achieve volumization and skin regeneration effects [16,55]. The reported advantages of micro fat grafting include enhanced safety in the periocular area [56], the ability to inject the fat superficially using syringe needles [21,56,57,58], and a higher content of stem cells [20].

Many studies have demonstrated that the smaller the injected adipose clusters, the more superficial tissue can be harvested, and the better the obtained results [16,20,21].

Moreover, when small (0.3 to 0.8 mm) adipose tissue clusters are injected, cellular blood irroration is improved, along with the degree of engraftment [30]. Coleman [55] emphasized that when placing fat, it is imperative to maximize the surface area contact with the surrounding tissue to ensure the proximity of the grafted fat to the recipient’s vasculature. Larger fat globules undergo central necrosis and volume loss and may result in oil cysts [58,59].

In recent years, regenerative antiaging medical treatment has attracted considerable interest from physicians and appreciation from patients, as this medical procedure is a natural therapy for tissue aging, and not merely a cosmetic treatment. The results are natural, without overfilled and fake effects. Many studies have proven the benefits of injecting adipose tissue, SVF, ADSCs, and GFs in aging tissue [60,61].

In light of this evidence, regenerative medical treatment is becoming the main procedure used to cure aged tissue; many combined treatments including other aesthetic medical procedures, such as hyaluronic acid filler, suspension threads, synthetic calcium hydroxylapatite, botulin toxin, and microneedling, are under investigation to improve the results and safety. The rationale for combining treatments is to perform the aesthetic treatment in a tissue with increased trophism and improved circulation.

In recent years, guided SEFFI has allowed for regenerative treatment to be carried out in many Italian aesthetic medical centers, and the number of patients who have undergone this treatment has been steadily increasing since 2019.

Thanks to the patented guide, guided SEFFI guarantees the collection of adipose tissue in the very superficial layer (SAT), which yields a higher amount of SVF cells [20,62].

Furthermore, the guide guarantees that the harvesting procedure is performed in a safe plane. The SEFFI cannula allows for the collection of adipose tissue that is already in small cellular clusters, avoiding any further mechanical manipulations while supporting high viability and stemness of the microfragmented adipose tissue [12,35].

The standardized technique for guided SEFFI use allows physicians without surgical skills to apply the guided SEFFI technique in a standardized, simple, safe, and effective way. This treatment enables physicians (not only surgeons) to accomplish regenerative therapy in aesthetic medicine for antiaging treatment by exploiting the capacity of SVF, ADSCs, and GFs that are naturally present in the adipose tissue [36].

In this study, we focused our attention on the safety of guided SEFFI and combined procedures.

Autologous fat transfer has many beneficial qualities that make it advantageous for the regeneration of soft tissue for reconstructive or cosmetic indications. Fat grafts are autologous, biocompatible, permanent, and viable when integrated into the injection site. They are easily accessible in most patients and represent a relatively simple and low-cost surgical procedure. The results of many studies provide more complete evidence describing the effectiveness and safety of autologous fat grafting in addition to illustrating the versatility of fat grafting and highlighting important details such as complication rates. A recent systematic review of complications in facial fat grafting including a total of 5479 patients reported 298 adverse events. The average complication rate ranged from 1.5% to 81.4%. A total of 298 adverse events were identified as follows: 40 (13.4%) intravascular injections, 13 (4.3%) cases of asymmetry, 57 (19.1%) irregularities, 22 (7.4%) cases of graft hypertrophy, 21 (7%) cases of fat necrosis, 73 (24.5%) prolonged edemas, 1 (0.3%) infection, 6 (2%) cases of prolonged erythema, 15 (5%) cases of telangiectasia, and 50 (16.8%) cases of acne activation. Side effects related to racial fat grafting can be grouped into the following three categories: severe, moderate, and minor. Severe (13.4%) side effects, such as intravascular injection or migration, require neurological or neurosurgical management and often lead to permanent disability or death. Moderate (38.3%) side effects, such as fat hypertrophy, necrosis, cyst formation, irregularities, and asymmetries, require a retouch operation. Minor (48.3%) side effects, such as prolonged edema or erythema, require no surgical management [63]. The results of our extensive, precise, and well-conducted study prove that guided SEFFI is a safe procedure. In our study that included a total of 2365 patients, we did not report any severe or moderate side effects—only minor side effects, i.e., ecchymosis (occurring both in donor and injection sites; 14.2% and 38.6%, respectively). Other complications occurred very infrequently, such as hematoma or skin irregularities lasting more than 20 days, which were experienced by only 1.3 and 0.2% of the patients, respectively. The results prove that these procedures are safe in light of evidence that the only minor complication was the appearance of ecchymoses at the donor and injection sites, which was a rare event in all harvesting and injection procedures. The rate of other complications was also low. Moreover, the shallow rate of complications at the harvesting site can be attributed to the small amount of harvested tissue relative to that collected in other harvesting or liposuction procedures previously described by other authors [44,63,64].

Regarding the harvesting area, we found that the abdomen was the first choice, as it was selected in 52% of patients, followed by the trochanteric site (in 32% of patients). Interestingly, the mean amount of tissue collected from both sites was 27.1 mL. Thus, this procedure is minimally invasive and allows for the collection of moderate amounts of tissue. Therefore, the investigated procedure can be safely performed in a medical facility under local anesthesia. After tissue harvesting and washing, the injection treatment was carried out in the whole face in almost one-quarter of the patients (23.6%). A single-area treatment was performed in fewer patients (18.4%). The most frequently treated area was the malar–zygomatic site (9.6%), where the average amount of injected tissue was 4.4 mL per side.

In this study, guided SEFFI was performed as a single treatment in almost two-thirds of the patients (62.7%). Of the remaining 37% of patients who received combined treatment, botulin toxin and hyaluronic acid were the most commonly selected techniques (used in 12.5% and 9.9% of patients, respectively).

This study also proves that the guided SEFFI procedure can be accompanied by a low rate of complications. However, more analyses on the use of guided SEFFI combined with other cosmetic procedures is a subject for future studies to corroborate the encouraging findings obtained in the present study. The results of this work will be published soon. This was a multicentric retrospective study, and the procedures were performed in private centers. We encountered some intrinsic difficulties in collecting homogeneous data in terms of patient selection and the evaluation of results, and we are aware that this aspect will have to be improved in future studies. In particular, selecting patients who were of homogeneous age and BMI and with similar life habits was difficult. Furthermore, although the guided SEFFI technique is a standardized procedure, personal in-tissue harvesting and injecting techniques may be possible.

## 5. Conclusions

The presented study proves that guided SEFFI alone or in combination with other facial rejuvenation techniques performed in medical facilities is a minimally invasive procedure with a shallow rate of complications. In light of this evidence, guided SEFFI is proven to be a promising medical antiaging treatment that combines effectiveness, safety, and simplicity.

## Figures and Tables

**Table 1:** Details of fat harvesting and injection sites used with zguided SEFFI technique. The number in parenthesis in the last column indicate the percentage of treated patients (total number of patients: N = 2365).

**Fat harvesting**	**Donor site**	**Avarage harvested volume (mL)**	**Number of patients (%)**
	Abdomen	27.1	1231 (52.1)
	Thocantheric	27.1	761 (32.4)
	Hip	22.6	296 (12.6)
	Knee	6.8	67 (2.8)
	Others	2.1	3 (0.1)
**SEFFI injection**	**Injection area**	**Avarage injected volume (mL)**	**Number of patients (%)**
Single area	Reriocular	1.4	63 (2.7)
	Temporal	1.1	5 (0.2)
	Malar-zygomatic	4.4	233 (9.9)
	Perioral–lip	1.3	34 (1.4)
	Jawline	2.8	91 (3.8)
	Other	5.2	14 (0.6)
	**Total**		440 (18.6)
Combined area	Full face	14.8	557 (23.6)
	Two areas	5.1	561 (23.7)
	Three areas	2.8	639 (27.0)
	Four areas	8.3	246 (10.4)
	**Total**		1935 (81.4)

**Table 2:** Details of the types of ART procedures performed using the guided SEFFI technique. The column on the right side of the table indicates the percentage of treated patients (total number of patients: N = 2365).

Guided SEFFI Procedures		Number of Procedures	Percentage of Patients
Single procedure		1484	62.7
Combined procedures	Botulin toxin	295	12.5
	Hyaluronic acids	233	9.9
	Threads	122	5.2
	Microneedling	85	3.6
	Calcium hydroxylapatite	65	2.7
	Carboxytherapy	51	2.6
	Endolift	24	1.0
	Other	0	0
	**Total**	**875**	**37.0**

**Table 3:** Summarized details of complications that occurred in the donor and injection sites of patients who underwent aesthetic rejuvenation treatment using the guided SEFFI technique.

Site of Complication	Type of Complication	Number of Events	Percentage of Patients
Donor site	Ecchymosis	336	14.2
	Hematoma	31	1.3
	Prolonged erythema (>48 h)	0	0
	Skin necrosis	0	0
	Prolonged edema (>20 days)	0	0
	Infection	0	0
	Fat necrosis	0	0
	Telangiectasias	0	0
	Skin irregularities (>20 days)	5	0.2
	Embolism	0	0
	Other	0	0
Injection site	Ecchymosis	914	38.6
	Hematoma	18	0.8
	Prolonged erythema (>48 h)	0	0
	Skin necrosis	0	0
	Prolonged edema (>5 days)	14	0.6
	Infection	1	<0.1
	Fat necrosis	0	0
	Telangiectasias	0	0
	Activation of acne	0	0
	Visibility	2	0.1
	Skin irregularities (>48 h)	1	<0.1
	Blindness	0	0
	Asymmetry	3	0.1
	Other	0	0

## Data Availability

The data presented in this study are available upon request from the corresponding authors. The data are not publicly available in order to respect the patients’ privacy.
